# Prevalence and radiographic features of cyst-like lesions in German Warmblood horses presented for pre-purchase examination

**DOI:** 10.3389/fvets.2026.1809383

**Published:** 2026-07-06

**Authors:** Elisabeth M. Wendling, Maren Hellige, Fritjof Freise, Philipp Lingens, Stefan Tietje, Uta Delling

**Affiliations:** 1 Clinic for Horses, University of Veterinary Medicine Hannover, Foundation, Hannover, Germany; 2 Department for Biometry, Epidemiology and Information Processing, University of Veterinary Medicine Hannover, Foundation, Hannover, Germany; 3 Equine Hospital Ankum, Ankum, Germany

**Keywords:** equine, joint, osteochondral conditions, purchase evaluation, radiology

## Abstract

**Introduction:**

Cyst-like lesions (CLLs) are frequently detected during radiographic examinations of equine limbs. They may represent a potential risk factor during pre-purchase examination (PPE) and are of considerable clinical and economic importance, as they may lead to negative purchase decisions. While affected horses may develop lameness, incidental findings have also been reported. The goal of the study was to describe the prevalence, location and radiographic characteristics of CLLs in a group of sound German Warmblood horses.

**Methods:**

A retrospective cohort study was conducted on 3863 German Warmbloods presented sound for pre-purchase examination at a German equine hospital over a five-year period. Radiographs were analyzed for CLL presence, and the age, gender, and breeding area (studbook) of horses affected by CLLs were documented. The CLLs were then independently evaluated by two observers using a novel comprehensive scoring system.

**Results:**

A total of 176 CLLs were detected in 164 horses revealing a CLL prevalence of 4.25%. The median age of the horses was 3.5 years, 67.68% of horses were male, and horses were mainly from Northwest Germany's studbooks. The most common location for CLLs was the proximal phalanx (32.96%) followed by the medial/lateral femoral condyles (16.48%). Hindlimbs were affected more frequently (64.77%). There was relatively high inter-observer agreement on the scoring system parameters, ranging from almost perfect (κ > 0.9) to moderate (κ = 0.5). Concomitant osteoarthritic changes of the affected joints were significantly associated with abaxial location of the CLL.

**Discussion:**

The overall prevalence of CLLs aligns with previous literature. Median age and higher proportion of males may reflect a selection bias for stallions undergoing licensing. The new scoring system showed excellent inter-observer agreement for objective parameters, subjective parameters yielded lower agreement. The retrospective design introduces potential underdiagnosis. The lack of caudocranial stifle projections in some PPE likely led to false negatives for medial femoral condyle lesions.

## 1 Introduction

Osseous cyst-like lesions (CLLs) are commonly encountered radiographic findings in horses. They are most commonly located close to the equine joint, though other locations within the bone are also described. Several pathogenetic mechanisms have been proposed, as they are either a manifestation of developmental (juvenile osteochondral condition, JOCC) or degenerative conditions (1). Further, JOCC is described as a multifactorial condition with both environmental (nutrition, exercise) and genetic factors having an impact (2–5).

CLLs are known to cause lameness and as such, have been subject of several previous publications (6–9). On the contrary, CLLs are also described as incidental finding in sound horses (10, 11). Importantly, CLLs are ranked as a risk factor for lameness in the German Equine Veterinary Association radiographic guidelines (12). As a consequence, CLLs detected during a pre-purchase examination (PPE) might result in negative purchase decisions due to a presumed risk for lameness, which in turn leads to economic loss (13).

There is little information available regarding the prevalence of CLLs in populations of warmbloods. In one study which focused on JOCC prevalence in general, rather than CLLs in particular, radiographic sets of weanlings of different breeds were evaluated as a part of the “Breeding, Osteochondral Status and Athletic Career program” in Normandy (France; *n* = 392). Selle Français (40.44%) were significantly more affected by JOCC compared to Thoroughbreds (24.23%) and French Trotters (35.32%) (14). A study about radiographic findings in PPE of warmbloods (*n* = 660) revealed an overall CLL prevalence of 3.6%, whereas the medial femoral condyle was affected most commonly (2.4%) (15). Studies in Thoroughbreds report prevalences ranging from below 1% in specific joints at yearling sales (16) to 11.1% in weanlings with radiographic findings, although precise lesion location is not provided (17). Lower prevalence (2.73%) has been described when investigation is focused on one specific region, such as the hindlimbs (18). Overall, the heterogeneity in CLL prevalence combined with the limited availability of breed-specific data in warmbloods underscores the gap of systematically comparable prevalence estimates.

There are several possible radiographic criteria characterizing a CLL appearance, including shape or the presence of a sclerotic rim (10, 19). Previous studies applying a radiographic grading system for CLLs focused on a single location only, i.e., the medial femoral condyle (20) or the distal aspect of the proximal phalanx (14). In one study comparing the visibility and radiographic features of CLLs diagnosed by computed tomography vs. radiography, several characteristics were defined that can be used to classify CLLs in every location of the equine limb, however, size of the CLLs was not included into the grading (21). In a retrospective study that surveyed a treatment option (Benzopyrone) for horses diagnosed with lameness due to CLL, a measurement system for CLLs was developed and three categories were established (< 15%, 15%−30%, >30% size of CLL in relation to the affected bone) (22).

To our knowledge, no previous studies have focused specifically on sound German Warmbloods with CLLs. Further, there is no evaluation score available to fully assess CLLs independent of their location within the equine skeleton. The objective of this study was therefore 1) to determine the prevalence of CLLs in a group of sound Warmbloods and 2) to develop and evaluate a CLL scoring system applicable for CLLs in various bones. Our hypotheses were, first, that the prevalence of CLLs in sound German Warmbloods is comparable to previous published numbers in other breeds, i.e., Thoroughbreds, and secondly, the score developed in the present study is reliably, proven by a high inter-observer agreement.

## 2 Materials and methods

### 2.1 Study design and population

A retrospective observational study was conducted using radiographic examinations obtained during pre-purchase examinations (PPE) at the Equine Hospital Ankum, Germany, between January 2012 and December 2016. To be included in the study, the horses needed to be sound during orthopedic examination and registered at a German Warmblood studbook.

The digital radiographic sets of each horse had to contain a minimum of 12 views, which strictly adheres to the applicable standard given by German Equine Veterinary Association for the period in question:

- lateromedial views of both front and hind distal limbs (all phalanges included)
- dorsolateral-plantaromedial oblique (DLPMO) view of the tarsus
- dorsomedial-plantarolateral oblique (DMPLO) view of the tarsus
- lateromedial view of the stifles
- upright pedal view of both front feet

Horses with incomplete radiographic sets or radiographs from referral veterinarians were excluded from the study. Other exclusion criteria were additional radiographic risk factors for lameness, e.g., large intraarticular osteochondral fragments or severe signs of osteoarthritis, in accordance with the PPE radiographic assessment guide of the German Equine Veterinary Association (12) current at the time of evaluation for the present study.

A veterinary practice management system (VetZ Easy Vet Software) was used to identify the cases and the integrated DICOM-Viewer EasyImage was subsequently used to evaluate the radiographic images for CLLs and other findings.

The following information were recorded for each horse that met the inclusion criteria: years of age at time of PPE, studbook (breeding area), sex, affected limb (front/hind, left/right), location within the bone (e.g., distal aspect of proximal phalanx).

### 2.2 Radiographic evaluation

The radiographs were assessed for findings in general and for CLLs by an equine surgeon (EW). CLLs were defined as well-demarcated “round radiolucency” according to the radiographic guidelines of German Equine Veterinary Association (12). All horses categorized as radiographic evident or suspected CLLs were assigned to the study group in the first step. Following, those radiographs were reviewed by two board-certified equine specialists (MH, UD) using a DICOM-viewer.^1^ Consensus was reached in case of disagreement.

**Figure 1:**
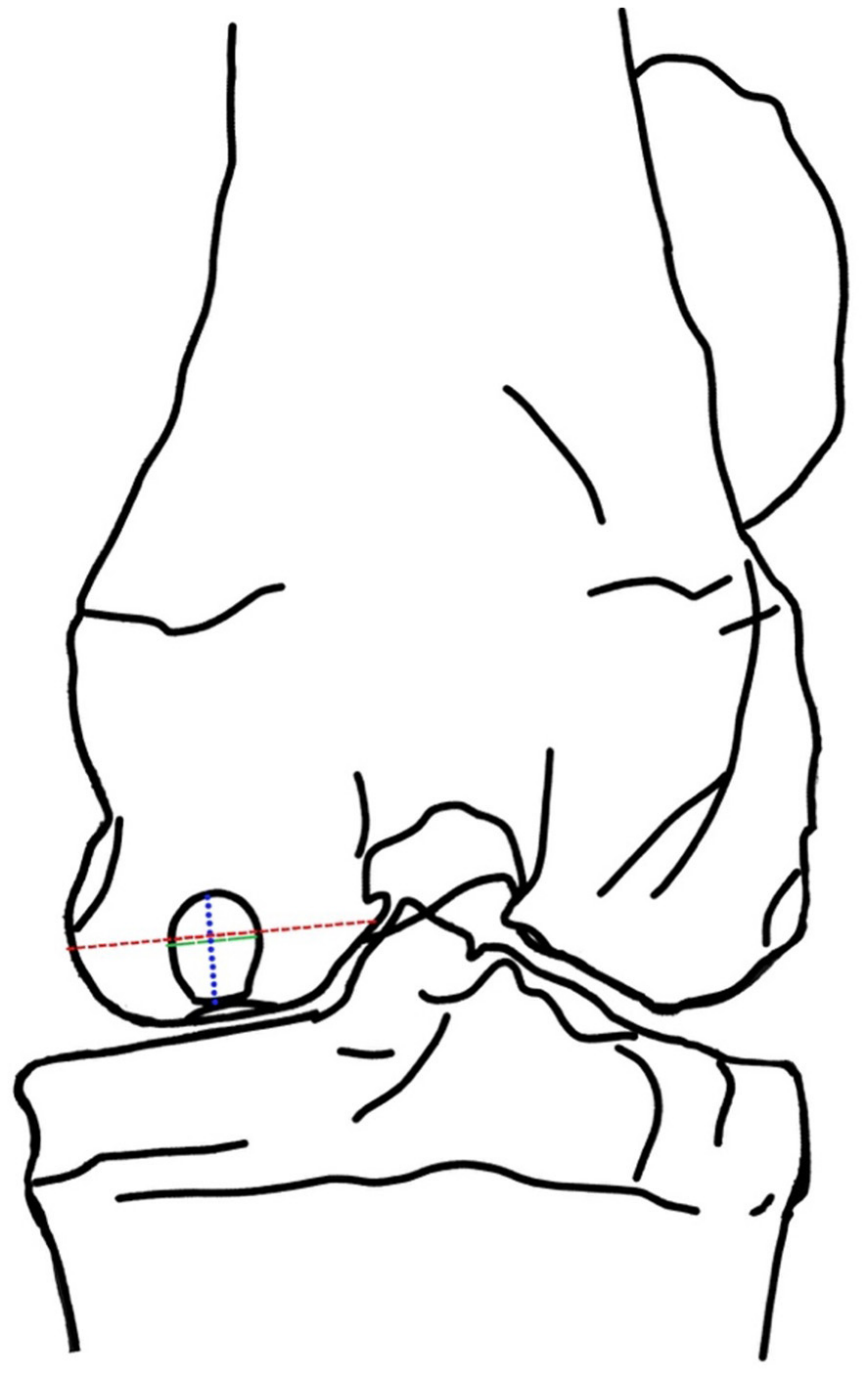
Measurements of the CLLs size depicted exemplary in the medial femoral condyle. CLL ratio = $\frac{horizontal\;diameter\;\left(green\;line\right)+vertical\;diameter\;(blue\;line)}{diameter\;affected\;bone\;(red\;line)}$ = [1 (< 0.15); 2 (0.15–0.3); 3 (>0.3)].

**Figure 2:**
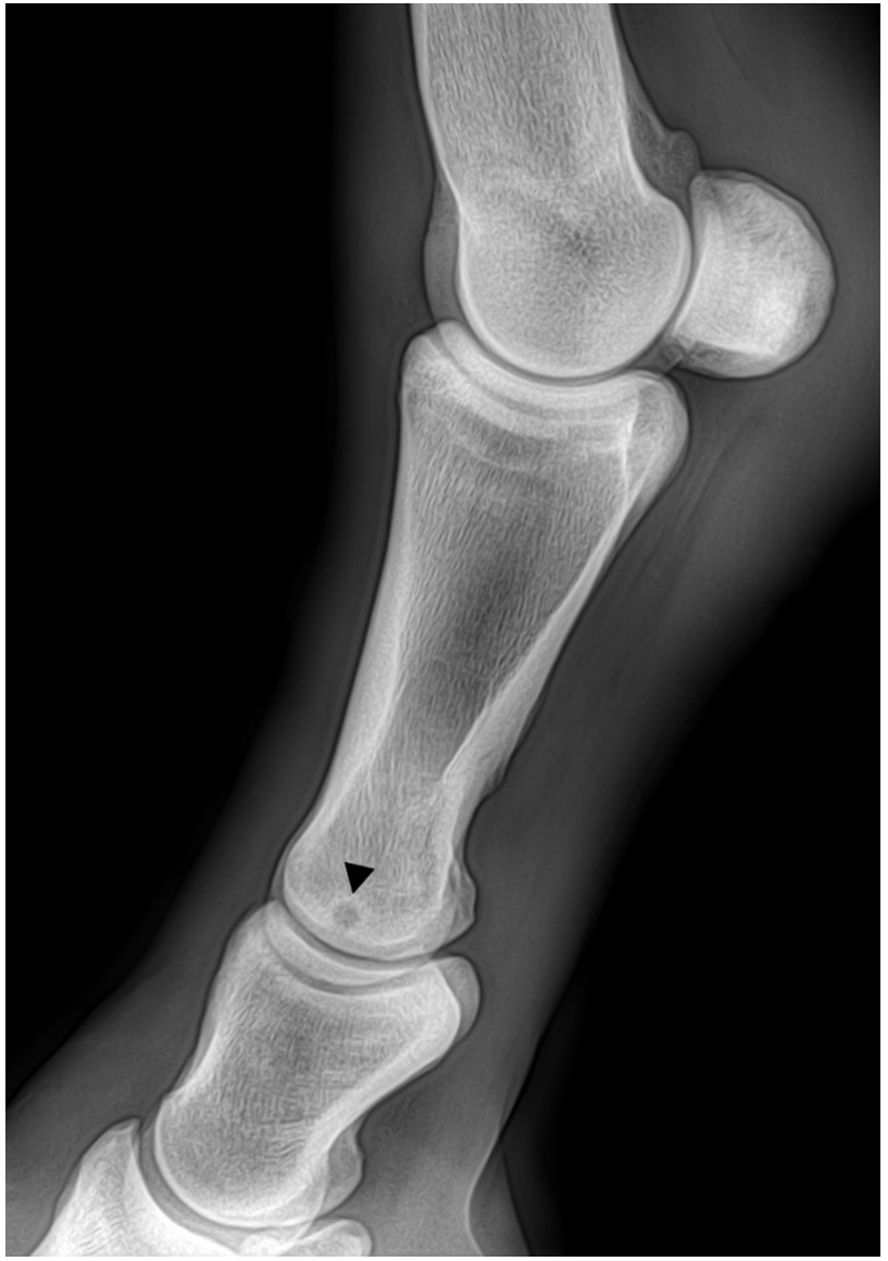
Lateromedial view of a distal limb: Example of a CLL (black arrowhead) in the distal subchondral bone of the proximal phalanx, example for an obvious sclerotic rim of CLL.

**Figure 3:**
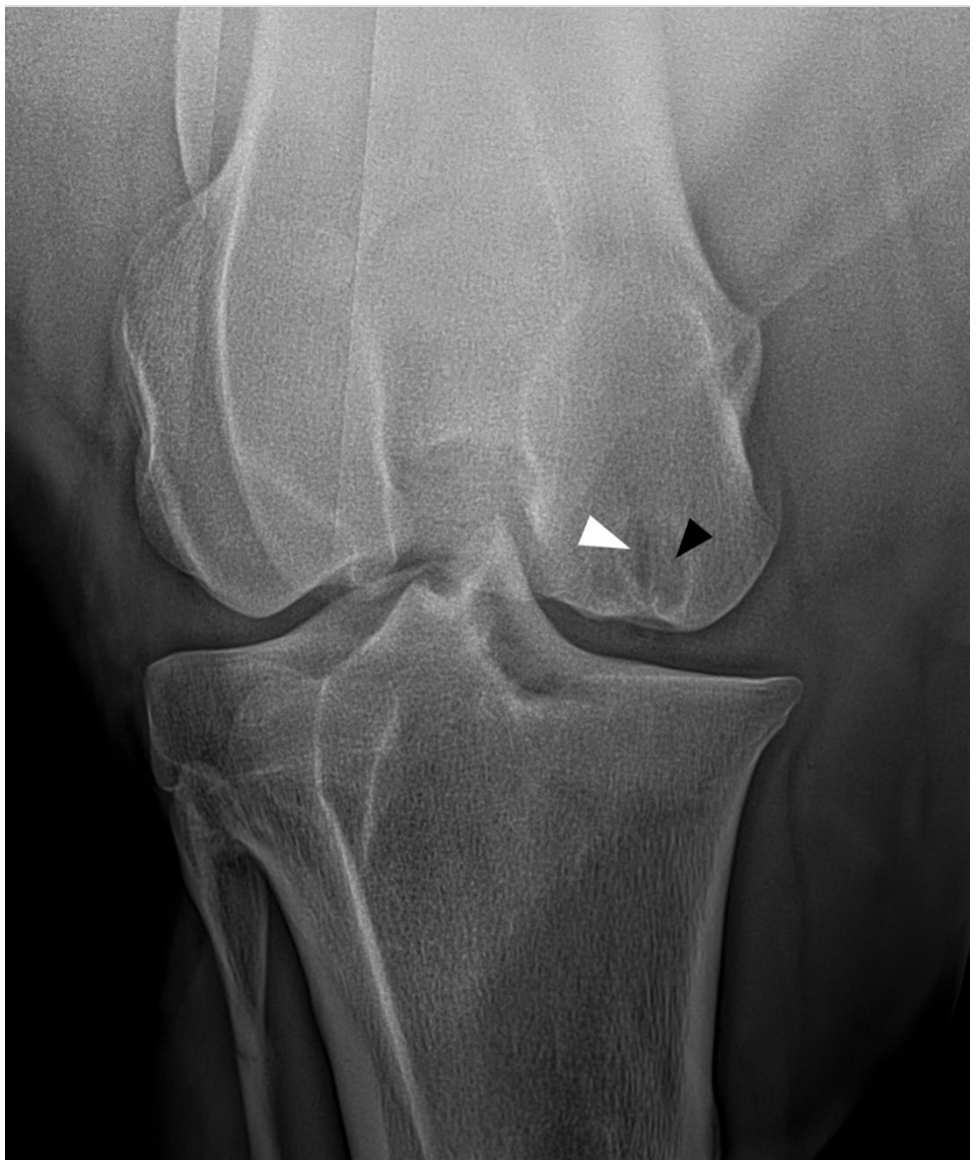
Caudocranial view of a stifle: Example of a heterogeneous CLL and present cloaca. Note the difference of radiopacity of the areas (white and black arrowheads).

**Figure 4:**
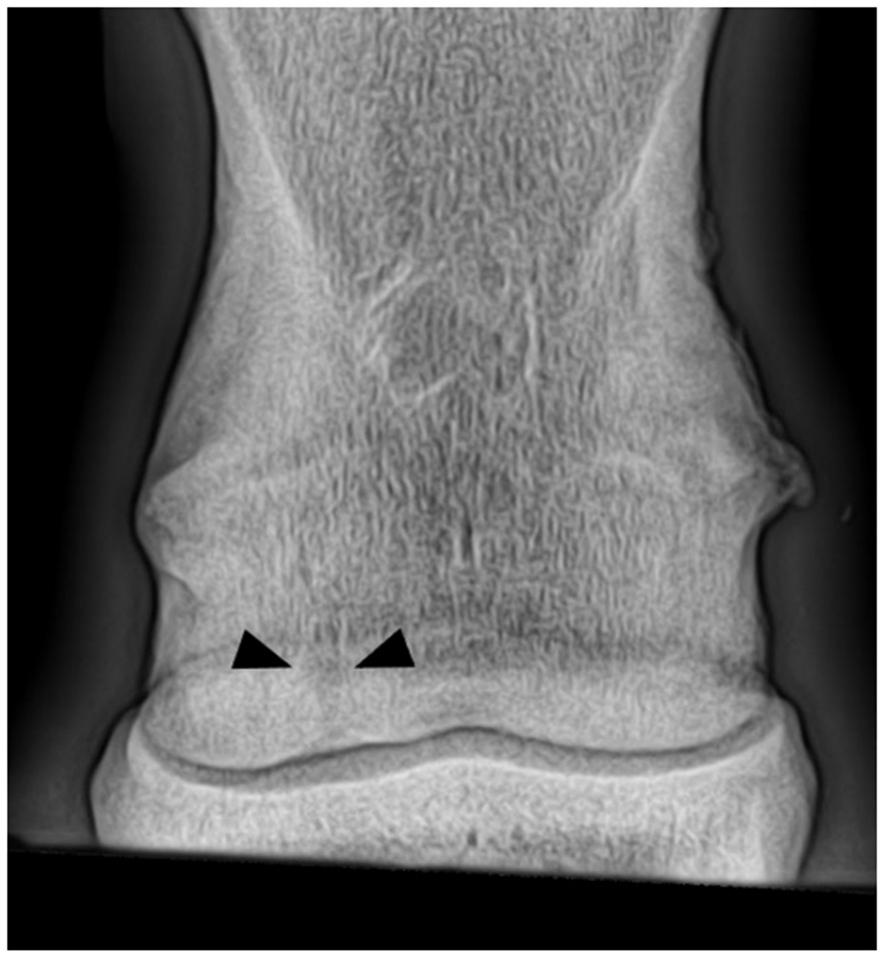
Dorsopalmar view of a distal limb: Example of an abaxial localization of CLL in the distal epiphysis of the proximal phalanx (black arrowheads).

**Figure 5:**
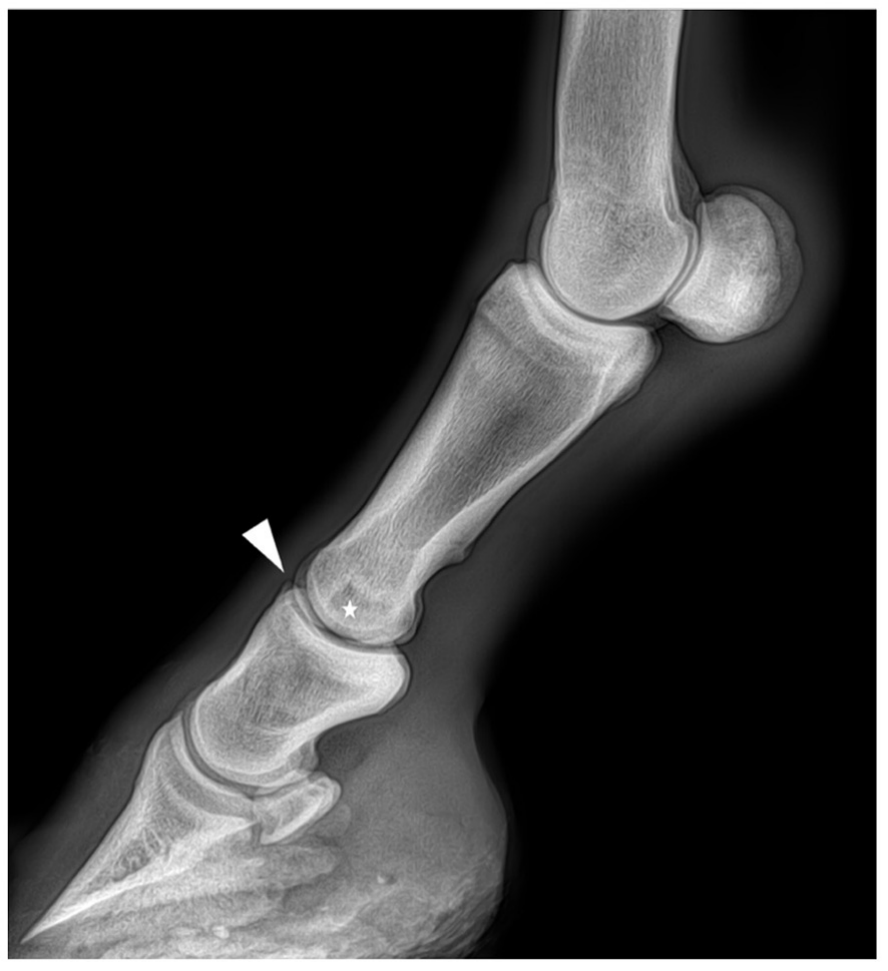
Lateromedial view of a distal limb: Examples for the presence of osteoarthritic changes: CLL (white asterisk) and osteophytes (white arrowhead).

**Figure 6:**
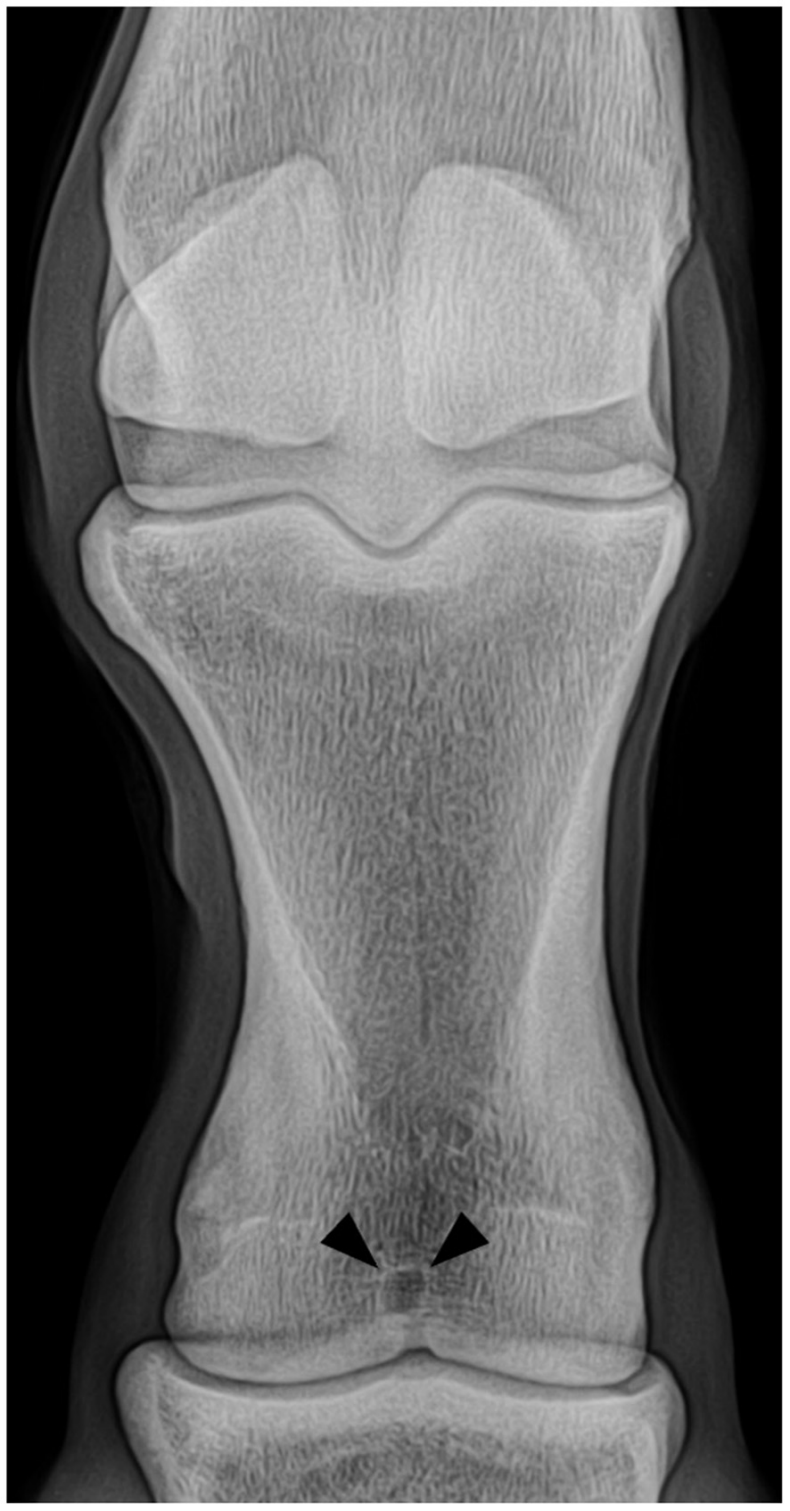
Dorsopalmar view of a distal limb: Example for a round shaped CLL in the distal and axial aspect of proximal phalanx (black arrowheads).

**Figure 7:**
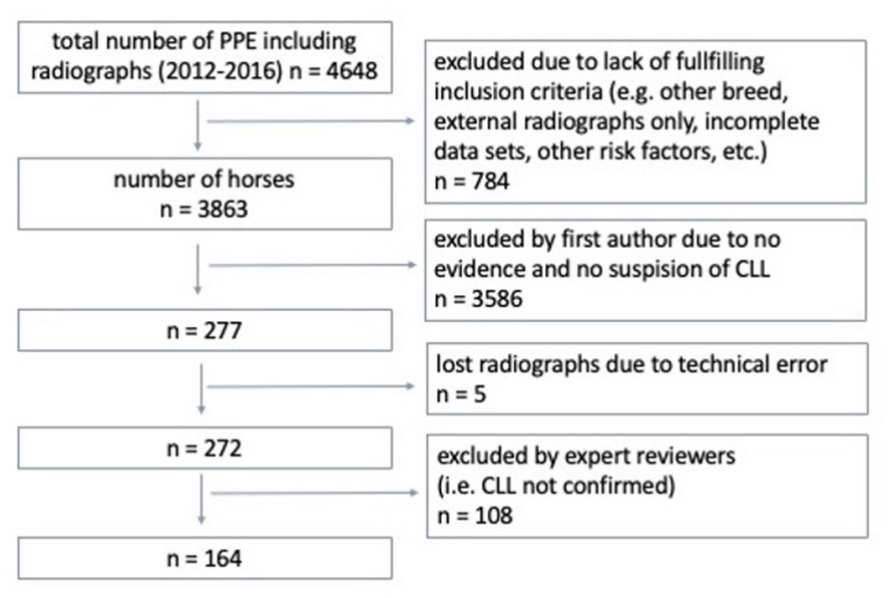
Flowchart on the inclusion of horses with CLLs into the present study.

**Figure 8:**
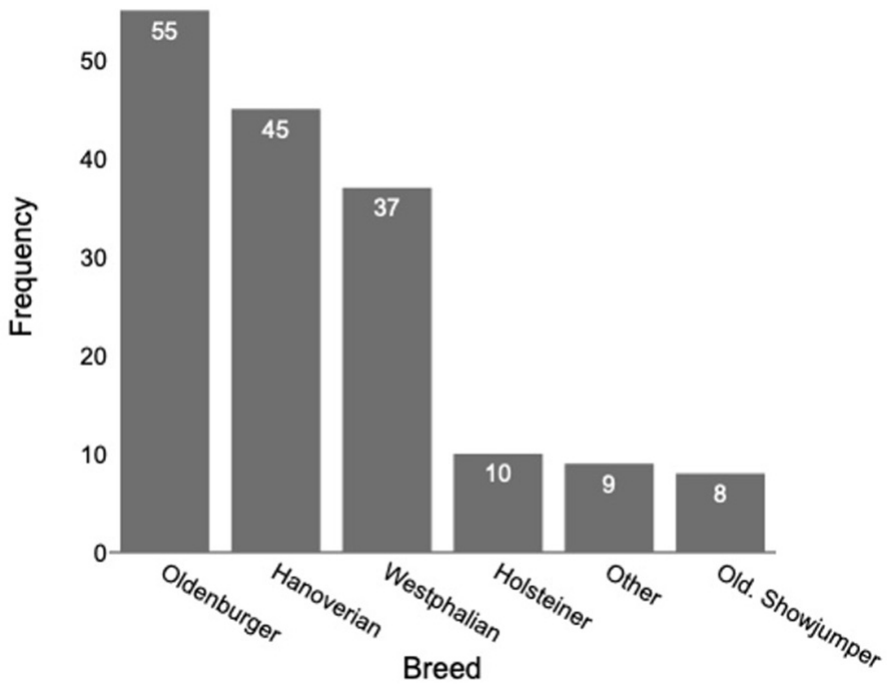
Distribution of Warmblood breeding areas (studbook) affected by cyst-like lesions within their skeletal bones (*n* = 164).

As a next step, all confirmed CLLs were further characterized. For that, previously published scoring systems were adapted (21, 22). The aim was to create a CLL score, which can be utilized for every location of CLL in the equine limbs (Table 1). The following criteria were included in the CLL score: (1) size ratio in relation to the affected bone (< 0.15, 0.15–0.3, >0.3; Figure 1) was measured in the dorsopalmar/dorsoplantar/caudocranial view if that projection was included in the radiographic set, otherwise the lateromedial view was used. To measure the size ratio, the vertical and horizontal diameters of the CLL were added and divided by the diameter of the affected bone, (2) subchondral-epiphyseal location (yes, no; Figure 2), (3) homogeneity/structure (homogeneous, inhomogeneous; Figure 3), (4) localization in the epiphysis (axial, abaxial; Figure 4), (5) sclerotic rim (absent, obscurely, obvious; Figure 2), (6) presence of osteophytes or other signs of osteoarthrosis (no, yes; Figure 5), (7) shape (round, oval, dome-shaped, shapeless; Figure 6), and (8) visible communication to the adjacent joint space / cloaca (yes, no; Figure 3). All radiographic images with CLLs were scored by two equine surgeons independently (EW, MH) using the aforementioned scoring system.

**Table 1:** The modified scoring system used in the present study to evaluate all CLLs of the equine limbs.

Scoring parameter	Evaluation criteria	Scale/characteristics	Example
Size	Size of lesion in relation to the affected bone	1/2/3	Figure 1: example for measurements
Subchondral-epiphyseal location	Location of the CLL in relation to the bone	Yes/No	Figure 2: subchondral
Homogeneity/Structure	Structure of the CLL	Homogeneous/Heterogeneous	Figure 3: heterogeneous
Localization in the epiphysis	Position of the CLL in relation to the axis	Axial/Abaxial	Figure 4: abaxial
Sclerotic rim	Presence of a sclerotic rim	Absent/Obscure/Obvious	Figure 2: obvious
Osteoarthritis	Signs of degenerative joint disease (e.g., osteophytes)	No/Yes	Figure 5: yes
Shape	Shape of the CLL	Round/Oval/Dome-Shaped/Shapeless	Figure 6: round
Cloaca	Presence of communication to the adjacent joint	Yes/No	Figure 3: present colaca

### 2.3 Data analysis

All data collected were transferred to Excel [Microsoft Excel, version 16.95 (Microsoft Corporation, Redmond, WA, USA)]. Statistical analysis was performed using the web-based program numiqo.^2^ Data sets were tested for normal distribution using the visual inspection of Q-Q plots and formally confirmed with a Shapiro–Wilk test. Data were presented as mean [95% confidence interval (CI), standard deviation (SD)] and median (IQR; Table 2). The Mann–Whitney *U*-test was applied if data not being normally distributed and was therefore used for comparison of age at time of CLL diagnosis between horses with and without signs of osteoarthrosis, the left/right and the front/hind distribution of CLLs. A logistic regression (Likelihood-Ratio-Test) was conducted in order to ascertain if sex influences front/hind distribution of CLLs. Furthermore, it was used to investigate the influence of size, shape and location of CLLs on signs of osteoarthrosis of the affected joint. The level of significance was set at *p* ≤ 0.05. To evaluate the inter-observer agreement of the CLL score, Cohens Kappa was applied (23).

**Table 2:** Detailed descriptive statistics of study population (^*^one of unknown sex).

Parameter	Studbook/Breed	Results		
Sex^*^		Male	*n* = 111	67.68%
		Female	*n* = 52	31.71%
Age at diagnosis		Mean	4.21	
Study population		SD	2.65	
		CI 95%	3.80–4.62	
		Median	3.5	
		IQR	4	
Age at diagnosis Studbook/Breed	Oldenburger	Mean	3.41	
		SD	3.02	
		CI 95%	2.65–4.17	
		Median	3	
		IQR	2	
	Hannover	Mean	3.73	
		SD	2.33	
		CI 95%	3.07–4.40	
		Median	3	
		IQR	3	
	Holsteiner	Mean	7.1	
		SD	2.13	
		CI 95%	5.58–8.62	
		Median	6.5	
		IQR	4	

## 3 Results

### 3.1 Study population

In total, 4,648 PPE including radiographic examinations were obtained between January 1st 2012 and December 31st, 2016. Of those, 3,863 horses fulfilled the inclusion criteria. Within this group, 277 horses had evidence or a suspicion of CLL; however, of those 108 cases were subsequently rejected and excluded from the study due to the review process and CLL not being confirmed by board certified specialists. In five cases, radiographs were lost due to a technical error. This resulted in 164 horses, which were included in the final CLLs study population (Figure 7). The PPE of those horses contained a minimum of 13 radiographic views to a maximum of 42 views (median 17).

The median age at time of PPE was 3.5 years (age ranged from 1 to 12 years; mean 4.21; Table 2). Data of age was not normally distributed (*p* < 0.001), the Q-Q plot shows a slight left skew. Overall, 31.00% (*n* = 51) of horses were 2 years of age or younger, and 55.00% (*n* = 90) were 3 years of age or younger.

There were 67.68% male and 31.71% female horses, in one horse sex was not documented. The studbook distribution of the examined horses indicated that 34.15% were Oldenburger, 27.44% were Hanoverian, and 21.95% were Westphalian (Figure 8), which were the three most frequent breeds.

Oldenburg and Hanoverian horses presented for PPE had the youngest median age with 3 years, while Holsteiners were presented with the oldest median age of 6.5 years.

### 3.2 Radiographs and cyst-like lesions

**Figure 9:**
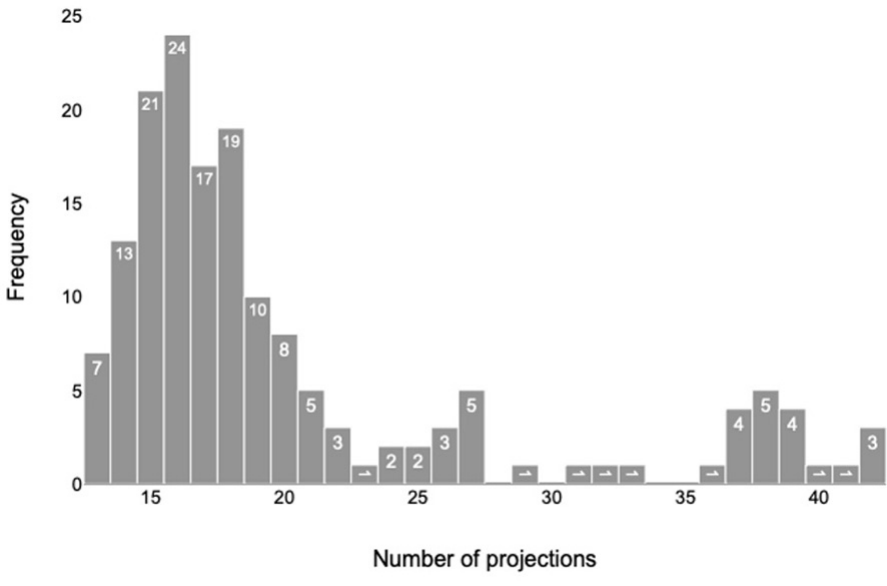
Distribution of the number of radiographic views per pre-purchase examination (minimum: 13, maximum: 42, median: 17).

Additional limb radiographs, beyond the required 12 views, were available for a large number of horses (Figure 9, minimum: 13, maximum: 42, median: 17).

In the 164 horses examined, a total of 176 CLLs were identified in five joints: distal interphalangeal joint (DIPJ), proximal interphalangeal joint (PIPJ), metacarpo-/metatarsophalangeal joint (McPJ/MtPJ), tarsus and stifle. Nine different bones were affected. Of the 164 horses, ten were found to have more than one CLL: in eight horses two CLLs were diagnosed in two different joints, one horse showed three CLLs in three different joints, and one horse presented with three CLLs in two different joints. In this horse, both stifles were affected with one CLL in the medial femoral condyle in the left and right hind limb each, and one CLL in the left hind proximal tibia. Therefore, only one horses was found with two CLLs in one joint (stifle), all other were one single CLL within one joint.

**Figure 10:**
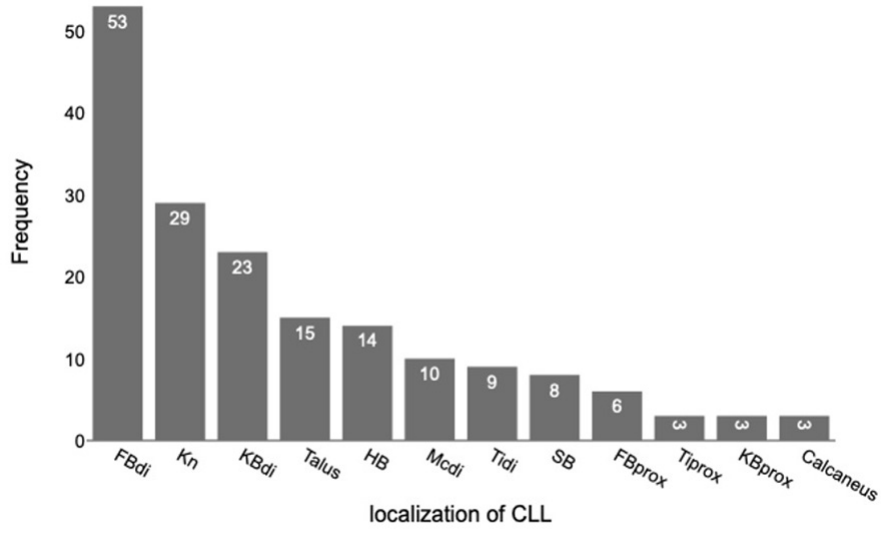
Distribution of the localizations of CLL within the skeletal bones, *n* = 176 FB: P1, KB: P2, HB: P3, SB, navicular bone; Mc, Metacarpus; Ti, Tibia; Kn, femoral condyle; di, distal aspect; prox, proximal aspect.

The distribution of CLLs within the bones was as follows (Figure 10): the most commonly affected bone was the proximal phalanx with 58/176 CLLs (32.96%) followed by the femur (medial or lateral condyle) with 29/176 CLLs (16.48%). Notably, out of 58 CLLs located in the proximal phalanx, 53 CLLs were located in the distal epiphysis, the remaining five were identified in the proximal epiphysis. Of the 161/176 CLLs (91.48%) which were located subchondral-epiphyseal and therefore possibly being connected to the adjacent joint space, the most commonly affected joint was the proximal interphalangeal joint with 55/161 CLLs (34.16%), followed by the distal interphalangeal joint with 35/161 CLLs (21.73%), and the stifle with 32/161 CLLs (19.87%).

Notably, 62/176 cases of CLL were located in the forelimbs (35.23%), while 114/176 cases (64.77%) were found in the hind limbs. The logistic regression showed a significant relation of sex on front/hind distribution of CLLs (*p* = 0.023) whereas male individuals showed more likely CLLs in the hind limbs. Laterality indicated that 98/176 (55.68%) of the CLLs were on the right side, compared to 78/176 (44.32%) on the left side of the horse.

Overall, the prevalence of CLLs in this group of sound German Warmblood horses was 4.25% (164/3,863; 95% CI 3.65%−4.93%).

### 3.3 Cyst-like lesions score

Out of the eight parameters, the inter-observer agreement was almost perfect for “size” (κ = 0.92), “localization in the epiphysis” (κ = 0.98), “subchondral-epiphyseal” (κ = 0.88), and “osteoarthritis” (κ = 0.85). Agreement was substantial for the parameter “shape” (κ = 0.64), “homogeneity/structure” (κ = 0.74), “cloaca” (κ = 0.77) and moderate for the category “sclerotic rim” (κ = 0.51). Due to the higher qualifications of MH, the scoring by MH was used for the final evaluation of all CLLs, despite the inter-observer agreement being overall moderate to almost perfect.

The distribution of the individual score parameter in the group of 176 CLLs were as follows (Table 3): regarding size, there were 88 cases (50.00%) of size 2, 63 (35.80%) were size 3, and 25 cases (14.20%) were size 1. The majority of the CLLs (*n* = 161, 91.48%) were located in the subchondral-epiphyseal region. Regarding the homogeneity, 66.48% (*n* = 117) of the CLLs exhibit a homogeneous structure. The distribution of axial vs. abaxial CLL location was similar (53.41% axial, 46.59% abaxial). Further, 50.57% (*n* = 89) showed an obvious sclerotic rim, 38.64% (*n* = 68) an obscurely sclerotic rim, and 10.80% (*n* = 19) had no sclerotic rim. Notably, 82.95% (*n* = 146) of the affected joints did not exhibit any evidence of osteoarthritic changes. The shape most commonly detected was a round shape 39.77% (*n* = 70). The majority of cases (86.36%, *n* = 152) exhibited evidence of communication to the adjacent joint through a cloaca.

**Table 3:** Results of the CLL scoring characteristics (*n* = 176 CLLs).

Parameter	Scale/characteristics	Quantity (*n*)	Distribution (%)
Size	1 (< 0.15)	25	14.20
	2 (0.15–0.3)	88	50.00
	3 (>0.3)	63	35.80
Subchondral-Epiphyseal	Yes	161	91.48
	No	15	8.52
Homogeneity/Structure	Homogeneous	117	66.48
	Heterogeneous	59	33.52
Localization in epiphysis	Axial	94	53.41
	Abaxial	82	46.59
Sclerotic rim	Absent	19	10.80
	Obscure	68	38.63
	Obvious	89	50.57
Osteoarthritis	No	146	82.95
	Yes	30	17.05
Shape	Round	70	39.77
	Oval	51	28.98
	Dome-shaped	36	20.45
	Shapeless	19	10.80
Cloaca	Yes	152	86.36
	No	24	13.64

Within 30 horses with CLLs and additional osteoarthritic changes in the affected joints, 11.93% (*n* = 21/176) of the CLLs were located abaxial, whereas 5.11% (*n* = 9/176) were located axial. Logistic regression showed a significant influence of the location (axial/abaxial) of the CLL on signs of osteoarthritis (*p* = 0.004).

Looking at the combination of CLL size and additional osteoarthritic changes, it was found that 15.34% (*n* = 27/176) were size 2 or 3, and 1.70% (*n* = 3/176) were size 1. There was, however, no significant influence of the size of CLL on osteoarthritic changes of the corresponding joint (*p* = 0.565).

Finally, in the subset of cases having osteoarthritic changes in the affected joint, 5.68% (*n* = 10/176) each were either dome or oval shaped, 3.98% (*n* = 7/176) of the CLLs were found to be round-shaped, and 1.70% (*n* = 3/30) were shapeless. There was no significant influence of shape of CLL on signs of osteoarthritis (*p* = 0.158).

The result of statistical analysis showed that higher values in size were measured on the hindlimbs (size ratio mean = 2.32; 95% CI 2.19–2.44; SD = 0.7) compared to the forelimbs (size ratio mean = 2.03; 95% CI 1.88–2.18; SD = 0.6).

Although there was a small, negative association between size and age at diagnosis (as the size increases, the age at the time of diagnosis tends to decrease), the association was not statistically significant (*p* = 0.142).

The Mann–Whitney *U*-test showed no significant difference in age at the time of CLL diagnosis between those with and without osteoarthritic changes (*p* = 0.472). Although small variations were identified between the left/right and front/hind groups regarding age at diagnosis, no significant differences were detected (*p* = 0.567 left/right; *p* = 0.791 front/hind).

Logistic regression analysis demonstrated that the presence of cloaca is significantly associated with a subchondral-epiphyseal location of CLL (*p* < 0.001), whereas other score parameters do not show an independent association.

## 4 Discussion

The first objective of the present study was to examine the prevalence of CLLs in a population of German Warmblood horses that were presented clinically sound for PPE at a single equine hospital in Germany. Based on our data, we can accept our first hypothesis, i.e., a prevalence of 4.25% lies within the previously published numbers of < 1 and 11.4% (16, 17) and is quite similar to the prevalence of 3.6% in a German study about PPE of Warmbloods (15). The few data available and the wide range reported illustrates the difficulty comparing those numbers to one another. Further, the CLL prevalence in the present study was higher compared to a previous study conducted in the same equine hospital (January 2014–October 2015), where a prevalence of 1.7% was reported in 2,145 radiographic sets of PPEs. A possible explanation is that in the previous study all horses were included regardless of their breed (11).

It is known that JOCC, including CLL, may have a genetic component, and breed may therefore play an important role. In the present study 61.59% of the horses were of Oldenburger or Hanoverian studbook. The equine hospital is located in a main breeding area of those two Warmbloods. That may also explain the younger age of the horses from those two studbooks at the time for PPE compared to horses from other studbooks, for example, Holsteiners. It is important to note, that all studbooks in the current study are open studbooks, which precludes any comparison between them or even any conclusions thereof. It is also worth mentioning that the horses presented for PPE are usually young horses that are being prepared for sale or intended for sport use. The mean age (4.21 years) in the present study was higher compared to previous data in Thoroughbreds (17) and slightly lower to previous data in Warmbloods (6.4 years) (15). This may be because, unlike Warmbloods, Thoroughbreds are put to sport use at an earlier age. Therefore, they are also subjected to routine radiographic and pre-purchase examinations earlier in their life. Comparing mean ages to other groups, CLL etiology has to be considered in the present study, meaning that both, JOCC and acquired CLLs, were likely present. There were slightly more male (67.68%) compared to female horses affected with CLLs. To the best of our knowledge sex distribution has not been reported in other studies. A study on JOCC in Thoroughbred weanlings (*n* = 65) reported a quite similar sex distribution (76.5% male) (24). It is also possible that for some reason more male than female horses were presented for PPE, which distorts the sex distribution of horses affected with CLLs. Furthermore, not all mares in warmblood breeding undergo radiographic examination, unlike stallions that are being prepared for licensing. Therefore, the study population may not be representative of the entire German Warmblood population.

Interestingly, there was a higher percentage of CLLs found in the hindlimbs (64.77%) compared to the frontlimbs. However, more radiographs were obtained from those limbs due to the known distribution of CLLs and other radiographic abnormalities. It is important to reiterate, that in the present study, carpi were not included, and therefore, our data may not be comparable to studies on Thoroughbreds, in which radiographs of the carpi are usually obtained (14, 16). It is possible that the radiographic protocol differences between studies may have influenced the detection rates of the lesions. In the present study, the inclusion criteria required 12 specific radiographic projections, which may have varied the likelihood of identifying CLLs compared to PPE protocols including more, less or other radiographic views. In respect to the CLL location, the proximal phalanx was affected most commonly in the present study (32.96%), which is similar to previous numbers in sound horses in the same breeding area (37.84%) (11). Importantly, this differs from a Swiss study in horses with lameness due to CLLs, in which the medial femoral condyle was more commonly affected (45.8%) (25). According to the minimum radiographic views requested by the present study, CLLs of the medial femoral condyle must be considered false negatives if only a lateromedial view of the stifle was provided, which is a main methodological limitation of the present study.

The second objective of this study was to develop a radiographic score for CLLs that is usable for every location CLLs may occur and test the inter-observer reliability. The findings of the present study propose a reliable and easily implementable scoring system for CLLs in various locations on the equine limbs. The inter-observer agreement for the scoring system developed in this study was notably high, particularly for parameters such as size (κ = 0.92) and localization in the epiphysis (κ = 0.98). This suggests that the scoring system is reliable and can be effectively utilized in clinical practice to assess CLLs in equine patients. Thus, we can accept our second hypothesis. It is noticeable that subjective parameters such as sclerotic rim showed lower agreement than objective parameters such as measured data. Those categories showed greater potential for interpretation in comparison to a clear category such as localization in epiphysis (axial/abaxial). The inter-observer agreement was similar high for obvious findings, such as CLLs in another German study, in which radiographs were evaluated by multiple experienced veterinarians using a guideline. Excellent inter-observer agreement was achieved for the detection of a CLL in the medial femoral condyle in caudocranial stifle projections, with 97.6% of observers identifying the same finding (26). Notably, Hellige et al. reported substantially higher agreement for these CLLs than Jackson et al. (27), who found only moderate to good inter-observer agreement among four specialized veterinary radiologists. In the study of Jackson et al., there was better agreement when the findings were clearly visible and defined. Higher kappa-values may be explained by the clearer definition and clearer visibility of the findings in this study. The scoring parameters with relatively low kappa-values (shape, homogeneity/structure, sclerotic rim) are overall subjective ones with unclear clinical relevance to date. Additionally, the significant association between the subchondral location of CLL and the presence of a visible communication with the adjacent joint space (cloaca) is not unexpected. This finding is consistent with the study by Amman et al. (28), in which 100% of subchondral CLLs demonstrated a connection to the adjacent joint space on computed tomography (CT) imaging. The resolution of a CT scan is significantly higher, which suggests that the CLL visible on the radiographic projection may have been connected, although this is not discernible from the radiograph alone. Further separate evaluation of these two scoring parameters with respect to athletic performance does not appear to be meaningful. Therefore, a simplified approach focusing on the distinction between subchondral and non-subchondral lesions may be more appropriate. Based on further investigation looking at sports performance data in detail it might be recommended to exclude the subjective parameters shape, homogeneity/structure, sclerotic rim, and cloaca from the scoring system. The significant association found between the abaxial location of CLLs, and signs of osteoarthritic changes further emphasizes the importance of thorough radiographic evaluation during PPE, as lesions in certain locations may have a higher risk for future joint issues. To the best of our knowledge, no study has focused on the influence of the size or shape of CLL on osteoarthritis. In a study on a treatment option for CLLs with benzopyrone (lame horses, *n* = 19), no significant influence of size of CLL or signs of osteoarthritis on outcome was observed (22). Based on this we question the clinical relevance of the parameters size and shape and look forward to further evaluation of the score parameters regarding long-term performance data.

Limitations of the present study are the retrospective design which may have led to an initial underdiagnosis of horses with CLLs, resulting in false negative findings. The absence of additional radiographic views in some cases, especially the caudocranial projection of stifles in some cases, also have contributed to missed lesions. Further limitation is the lack of repeated radiographs of the CLL affected joints regarding prediction of progression and development of osteoarthrosis. Additionally, the study population exhibited considerable age variability, thus JOCC and acquired CLL are likely both present. Specific results for either etiology would be more desirable. Finally, the study group was predominantly composed of horses from Northern and Western German breeding regions, while other German Warmblood breeds were underrepresented. Further research should focus on applying the scoring system to a larger and more diversified cohort of horses. Additionally, the evaluation of the radiographic characteristics should be conducted by multiple observers with varying degrees of expertise, in order to validate its reliability.

Looking to the future, the scoring system could also be implemented within an artificial intelligence software for rating PPE radiographs. Beyond its applications in research, the proposed scoring system may also support clinical decision-making during pre-purchase examinations by providing a more standardized assessment of CLL characteristics. This could enhance communication among veterinarians, buyers, and owners, thereby facilitating more objective risk assessment regarding future athletic performance or the potential development of osteoarthritis. Additionally, the scoring system may promote the longitudinal monitoring of lesion progression, thereby facilitating the development of treatment recommendations and follow-up strategies for clinical patients. Furthermore, it would be interesting to investigate the prognosis of CLLs diagnosed in sound horses to be able to give a prospect in the situation of pre-purchase exams to the client.

Furthermore, elucidating the long-term clinical significance and athletic outcome of CLLs in sound horses represents a critical next step, as such evidence could substantially refine prognostic assessment during pre-purchase examinations and contribute to more evidence-based management and purchasing decisions.

## Data Availability

The raw data supporting the conclusions of this article will be made available by the authors, without undue reservation.

## Footnotes

1 Horos, www.horosproject.org.

2 numiqo Team (2026). numiqo: Online Statistics Calculator. numiqo e.U. Graz, Austria. https://numiqo.de.

## References

[1] Semevolos SA. Osteochondritis dissecans development. Vet Clin North Am Equine Pract. (2017) 33:367–78. doi: 10.1016/j.cveq.2017.03.009 28551287

[2] Hurtig MB Pool R. Pathogenesis of equine osteochondrosis. In: McIlwraith CW Trotter GW, editors. Joint Disease in the Horse. Philadelphia, PA: W.B. Saunders Company (1996). p. 335–58.

[3] van Weeren PR Denoix JM. The Normandy field study on juvenile osteochondral conditions: conclusions regarding the influence of genetics, environmental conditions and management, and the effect on performance. Vet J. (2013) 197:90–5. doi: 10.1016/j.tvjl.2013.03.047 23639367

[4] Trotter GW McIlwraith CW. Osteochondritis dissecans and subchondral cystic lesions and their relationship to osteochondrosis in the horse. J Equine Vet Sci. (1981) 1:157–62. doi: 10.1016/S0737-0806(81)80029-9

[5] Stock KF Distl O. Survey on the development of Hanoverian Warmblood horses selected for sale at auction in 1991 to 1998. J Equine Vet Sci. (2005) 25:210–23. doi: 10.1016/j.jevs.2005.04.004

[6] Ravanetti P Lechartier A Hamon M Zucca E. A composite absorbable implant used to treat subchondral bone cysts in 38 horses. Equine Vet J. (2022) 54:97–105. doi: 10.1111/evj.13428 33502044

[7] Wallis TW Goodrich LR McIlwraith CW Frisbie DD Hendrickson DA Trotter GW . Arthroscopic injection of corticosteroids into the fibrous tissue of subchondral cystic lesions of the medial femoral condyle in horses: a retrospective study of 52 cases (2001-2006). Equine Vet J. (2008) 40:461–7. doi: 10.2746/042516408X258843 18089474

[8] Boorman S Hofmeister EH Ross MW Ralston S Bell G Mackie S . Influence of osteochondrosis on the longevity and racing performance of standardbred trotters and pacers. Vet Surg. (2021) 50:1–10. doi: 10.1111/vsu.13568 33460472

[9] Cohen ND Carter GK Watkins JP O'Conor MS. Association of racing performance with specific abnormal radiographic findings in Thoroughbred yearlings sold in Texas. J Equine Vet Sci. (2006) 26:462–74. doi: 10.1016/j.jevs.2006.08.004

[10] Butler J Colles C Dyson S Poulos P. Clinical radiology of the horse. In: Clinical Radiology of the Horse. 4th, ed. Chichester, Wiley Blackwell (2017). p. 55–148.

[11] Leonczuk B Tietje S Lingens P. Subchondral bone cysts/cyst-like lesions in radiographic screening of horses in purchase examinations. Praktische Tierarzt. (2016) 97:416–25.

[12] GPM. Röntgen-Leitfaden. Frankfurt: GPM (2018).

[13] Seitzinger AH Traub-Dargatz J Kane A Kopral C Morley P Garber L . A comparison of the economic costs of equine lameness, colic, and equine protozoal myeloencephalitis (EPM). In: Proceedings of the 9th international symposium on veterinary epidemiology and economics. Breckenridge, Co: International Society for Veterinary Epidemiology and Economics (2000). p. 1–3.

[14] Denoix JM Jacquet S Lepeule J Crevier-Denoix N Valette JP Robert C. Radiographic findings of juvenile osteochondral conditions detected in 392 foals using a field radiographic protocol. Vet J. (2013) 197:44–51. doi: 10.1016/j.tvjl.2013.03.040 23643868

[15] Markert S. Occurrence of findings of the 2007 radiology guideline in pre-purchase examinations in a German horse clinic and registration of the following performance of warmblood horses in competition. Stiftung Tierärztliche Hochschule Hannover, Hannover (2016).

[16] Kane AJ Park RD McIlwraith CW Rantanen NW Morehead JP Bramlage LR. Radiographic changes in Thoroughbred yearlings. Part 1: prevalence at the time of the yearling sales. Equine Vet J. (2003) 35:354–65. doi: 10.2746/042516403776014280 12880003

[17] Axling JM Castle K Velie BD Tammen I Thomson PC Hamilton NA . Use of diagnostic reports to estimate prevalence and distribution of skeletal lesions in young Thoroughbreds. Vet J. (2016) 214:72–6. doi: 10.1016/j.tvjl.2016.03.022 27387729

[18] Robert C Valette JP Jacquet S Denoix JM. Influence of juvenile osteochondral conditions on racing performance in Thoroughbreds born in Normandy. Vet J. (2013) 197:83–9. doi: 10.1016/j.tvjl.2013.03.046 23639369

[19] Sherlock C Mair T. Osseous cyst-like lesions/subchondral bone cysts of the phalanges: phalangeal osseous cyst-like lesions. Equine Vet Educ. (2011) 23:191–204. doi: 10.1111/j.2042-3292.2010.00222.x

[20] Jackson WA Stick JA Arnoczky SP Nickels FA. The effect of compacted cancellous bone grafting on the healing of subchondral bone defects of the medial femoral condyle in horses. Vet Surg. (2000) 29:8–16. doi: 10.1111/j.1532-950X.2000.00008.x 10653490

[21] Schön S Fürst AE Ohlerth S Kircher PR Roos M Jackson MA. Computed tomographic versus radiographic assessment of the visibility and features of subchondral cystic lesions in equine limbs. Pferdeheilkunde. (2017) 33:256–62. doi: 10.21836/PEM20170306

[22] Jackson MA Fricker-Feer C Kümmerle JM Fürst AE. Die Behandlung von subchondralen zystoiden Defekten beim Pferd mit Benzopyron: eine retrospektive Analyse. Wiener Tierärztliche Monatsschrift. (2008) 95:158–65. doi: 10.5167/UZH-13208

[23] Landis JR Koch GG. The measurement of observer agreement for categorical data. Biometrics. (1977) 33:159. doi: 10.2307/2529310 843571

[24] Bastos LFC Dubiella A Bastos FZ Barussi FCM Webber SH Costa MFDM . Incidence of juvenile osteochondral conditions in thoroughbred weanlings in the South of Brazil. J Equine Vet Sci. (2017) 54:12–7. doi: 10.1016/j.jevs.2017.02.008

[25] von Rechenberg B McIlwraith CW Auer JA. Cystic bone lesions in horses and humans: a comparative review. Vet Comp Orthop Traumatol. (1998) 11:08–18. doi: 10.1055/s-0038-1632602

[26] Hellige M Rohn K Buschkamp L Stadler P. Interobserver agreement in interpreting radiographs of horses using the German Radiology-Guidelines 2007 (“Röntgenleitfaden 2007”). Pferdeheilkunde. (2018) 34:212–22. doi: 10.21836/PEM20180301

[27] Jackson MA Vizard AL Anderson GA Mattoon JS Lavelle RB Smithenson BT . An assessment of intra- and interobserver agreement of reporting orthopaedic findings on presale radiographs of Thoroughbred yearlings. Equine Vet J. (2014) 46:567–74. doi: 10.1111/evj.12150 23889034

[28] Ammann L Ohlerth S Fürst AE Jackson MA. Differences of morphological attributes between 62 proximal and distal subchondral cystic lesions of the proximal phalanx as determined by radiography and computed tomography. ajvr. (2022) 83:ajvr.22.04.0071. doi: 10.2460/ajvr.22.04.0071 36315450

